# Brain network characteristics between subacute and chronic stroke survivors in active, imagery, passive movement task: a pilot study

**DOI:** 10.3389/fneur.2023.1143955

**Published:** 2023-07-19

**Authors:** Yifang Lin, Zewu Jiang, Gege Zhan, Haolong Su, XiaoYang Kang, Jie Jia

**Affiliations:** ^1^Department of Rehabilitation Medicine, Huashan Hospital, Fudan University, Shanghai, China; ^2^Department of Rehabilitation Medicine, Shanghai Jing’an District Central Hospital, Shanghai, China; ^3^Academy for Engineering and Technology, Fudan University, Shanghai, China; ^4^National Clinical Research Center for Aging and Medicine, Huashan Hospital, Fudan University, Shanghai, China; ^5^National Center for Neurological Disorders, Shanghai, China

**Keywords:** stroke stage, brain network topology, upper limb, electroencephalography, task-related coherence

## Abstract

**Background:**

The activation patterns and functional network characteristics between stroke survivors and healthy individuals based on resting-or task-state neuroimaging and neurophysiological techniques have been extensively explored. However, the discrepancy between stroke patients at different recovery stages remains unclear.

**Objective:**

To investigate the changes in brain connectivity and network topology between subacute and chronic patients, and hope to provide a basis for rehabilitation strategies at different stages after stroke.

**Methods:**

Fifteen stroke survivors were assigned to the subacute group (SG, *N* = 9) and chronic group (CG, *N* = 6). They were asked to perform hand grasping under active, passive, and MI conditions when recording EEG. The Fugl-Meyer Assessment Upper Extremity subscale (FMA_UE), modified Ashworth Scale (MAS), Manual Muscle Test (MMT), grip and pinch strength, modified Barthel Index (MBI), and Berg Balance Scale (BBS) were measured.

**Results:**

Functional connectivity analyses showed significant interactions on frontal, parietal and occipital lobes connections in each frequency band, particularly in the delta band. The coupling strength of premotor cortex, M1, S1 and several connections linked to frontal, parietal, and occipital lobes in subacute subjects were lower than in chronic subjects in low alpha, high alpha, low beta, and high beta bands. Nodal clustering coefficient (CC) analyses revealed that the CC in chronic subjects was higher than in subacute subjects in the ipsilesional S1 and occipital area, contralesional dorsolateral prefrontal cortex and parietal area. Characteristic path length (CPL) analyses showed that CPL in subacute subjects was lower than in chronic subjects in low beta, high beta, and gamma bands. There were no significant differences between subacute and chronic subjects for small-world property.

**Conclusion:**

Subacute stroke survivors were characterized by higher transfer efficiency of the entire brain network and weak local nodal effects. Transfer efficiency was reduced, the local nodal role was strengthened, and more neural resources needed to be mobilized to perform motor tasks for chronic survivors. Overall, these results may help to understand the remodeling pattern of the brain network for different post-stroke stages on task conditions and the mechanism of spontaneous recovery.

## 1. Introduction

Stroke is still the leading cause of disability in China with over 2 million new cases annually (1). Upper limb impairment and recovery are important for stroke survivors (2). Post-stroke motor impairment is closely associated with altered brain functions (3). Investigating the brain network characteristics of stroke survivors is one way to learn the underlying mechanisms of stroke recovery and rehabilitation. Numerous studies have investigated the characteristics of resting-or task-state functional networks between stroke survivors and healthy individuals to find new targets for stroke rehabilitation through functional magnetic resonance imaging (fMRI), electroencephalogram (EEG) or functional near-infrared spectroscopy (fNIRS) (4–7). In fact, comparing altered brain networks between stroke survivors and health control seems far from enough. There was an obvious discrepancy for stroke patients at different recovery stages. The time interval from stroke was considered a grouping factor to analyze its impact on the post-stroke recovery (8). Spontaneous recovery in the first six months after stroke (i.e., subacute phase) has been widely accepted (9–12). Consensus statements from the Stroke Recovery and Rehabilitation Roundtable suggested that individualized rehabilitation strategies should be developed for different timelines of stroke recovery (13). Thus, exploring changes in brain networks between subacute and chronic patients seems necessary.

Resting-state functional networks are valuable in understanding complex brain communication (14). Task-related connectivity is also essential to reflect the human brain’s ability to alter adaptively. Recently, the important role of task-related functional connections in dynamically reshaping brain network organization, shifting the flow of neural activity has been reported (15, 16). However, task-based fMRI or EEG tended to investigate activation patterns during task conditions through blood oxygen level-dependent (BOLD) response or event-related desynchronization (ERD) changes in previous research, which focused on a small fraction of overall brain activity (17, 18). Although several studies have probed neural networks that are involved in brain activation by dynamic causal modeling (DCM), DCM as a prior approach requires user-specified regions of interest (ROI) and is limited by model numbers (19, 20). Therefore, the number of regions of interest (ROIs) that can be used for DCM analysis is limited. Coherence is not limited by such factors and could be applied in both resting-state and task-state conditions, as well as seen as reflecting changes in the degree of coupling between areas (21). Graph theory approach is a very useful tool for exploring advanced neural networks. Human brain function networks typically have a high clustering coefficient (CC) and short characteristic path length (CPL), which is also known as small-world (SW) property. After stroke, the small-world model of the brain network usually decreased but was frequency-dependent in the EEG study (22). In addition, compared to EEG, fMRI is more susceptible to head movement. Maintaining head stability during tasks was a major challenge for stroke survivors. Meanwhile, patients performing motor tasks in daily treatment states are more convenient with EEG. Therefore, calculating network characteristics based on coherence and graph theory *via* EEG is a good choice.

Selection of task paradigms during EEG signal acquisition should be noted. Numerous task-based EEG studies have used active, passive, and motor imagery (MI) tasks alone, or two or all of them in study design (23–25). In fact, these task paradigms are commonly used in clinical practice. Active tasks included motor execution and motor attempt, in which the former required the subject to have a visible motor output and the latter required only an attempt to exercise. Therefore, motor attempts are more suitable for stroke survivors. Unlike active task, MI task is the cognitive rehearsal of specific actions without overt motor output (26). Active and MI task belonged to active-rehabilitation techniques, while passive task was as an auxiliary training strategy. In this study, participants were asked to perform hand grasping under active, passive, and MI conditions to fully understand changes in the task-induced neural network between subacute and chronic patients. Based on the current study, we hypothesize that neural resource recruitment of brain regions associated with the frontoparietal motor network for chronic stroke survivors was higher than for patients with subacute stroke when performing the different task with the affected hand, which may provide more insights into post-stroke motor recovery.

## 2. Method

### 2.1. Subjects

Survivors with hemiparesis and a radiologically confirmed stroke from inpatient services in Huashan Hospital were recruited. All subjects signed informed consent forms. Inclusion criteria were 25 to 75 years of age, unilateral ischemic or hemorrhagic stroke for the first time, and stroke that occurred more than 1 week. Survivors with psychiatric disorder, epilepsy, active malignant disease or multiple organ failure, excessive cognitive impairment, neglect, or apraxia and allergy to EEG electrode cream were excluded. The study was approved by the Institutional Review Boards of Huashan Hospital (KY2022-041). Using the above inclusion/exclusion criteria, 16 subjects were recruited. One subject’s EEG data was discarded due to large artifacts. They were assigned to two groups: the subacute phase group (*n* = 9, 7 males and 2 females) and the chronic phase group (*n* = 6, 4 males and 2 females) according to the stroke recovery timeline presented at the first Stroke Recovery and Rehabilitation Roundtable (13). Their demographic and clinical characteristics are shown in Table 1.

**Table 1:** Demographics data and clinical outcomes of stroke survivors.

Characteristics	SG	CG	*p*
Age, years, mean (SD)	63.11 (11.74)	54.00 (14.97)	0.586
Months after stroke onset, mean (SD)	2.40 (1.54)	59.64 (58.28)	<0.001*
Gender, N			1.000
Male	7	4	
Female	2	2	
Side of paralysis, N			0.622
Right	3	3	
Left	6	3	
FMA, Q2 (Q1, Q3)			
FMA_UE (max = 66)	19.00 (1.00, 55.50)	37.00 (9.25, 52.50)	0.595
FMA-hand (max = 24)	2.00 (0, 11.00)	7.00 (2.50, 11.75)	0.285
Grip strength, Q2 (Q1, Q3)			
Unaffected hand	24.70 (19.90, 25.95)	27.40 (17.50, 28.85)	0.205
Affected hand	3.20 (0, 11.00)	11.7 (1.20, 14.10)	0.408
Pinch strength, Q2 (Q1, Q3)			
Unaffected hand	5.20 (4.25, 6.90)	7.00 (5.05, 7.35)	0.254
Affected hand	0 (0, 1.20)	2.00 (0.95, 3.10)	0.152
MBI, Q2 (Q1, Q3)	94.00 (65.00, 97.50)	98.00 (26.00, 100.00)	0.789
BBS, Q2 (Q1, Q3)	52.50 (33.75, 56,00)	53.00 (31.50, 55.00)	0.552

BBS, berg balance scale; CG, chronic group; FMA_UE, fugl-meyer assessment upper extremity subscale; MBI, modified barthel index; SG, subacute group.

### 2.2. Experimental paradigm and tasks

**Figure 1:**
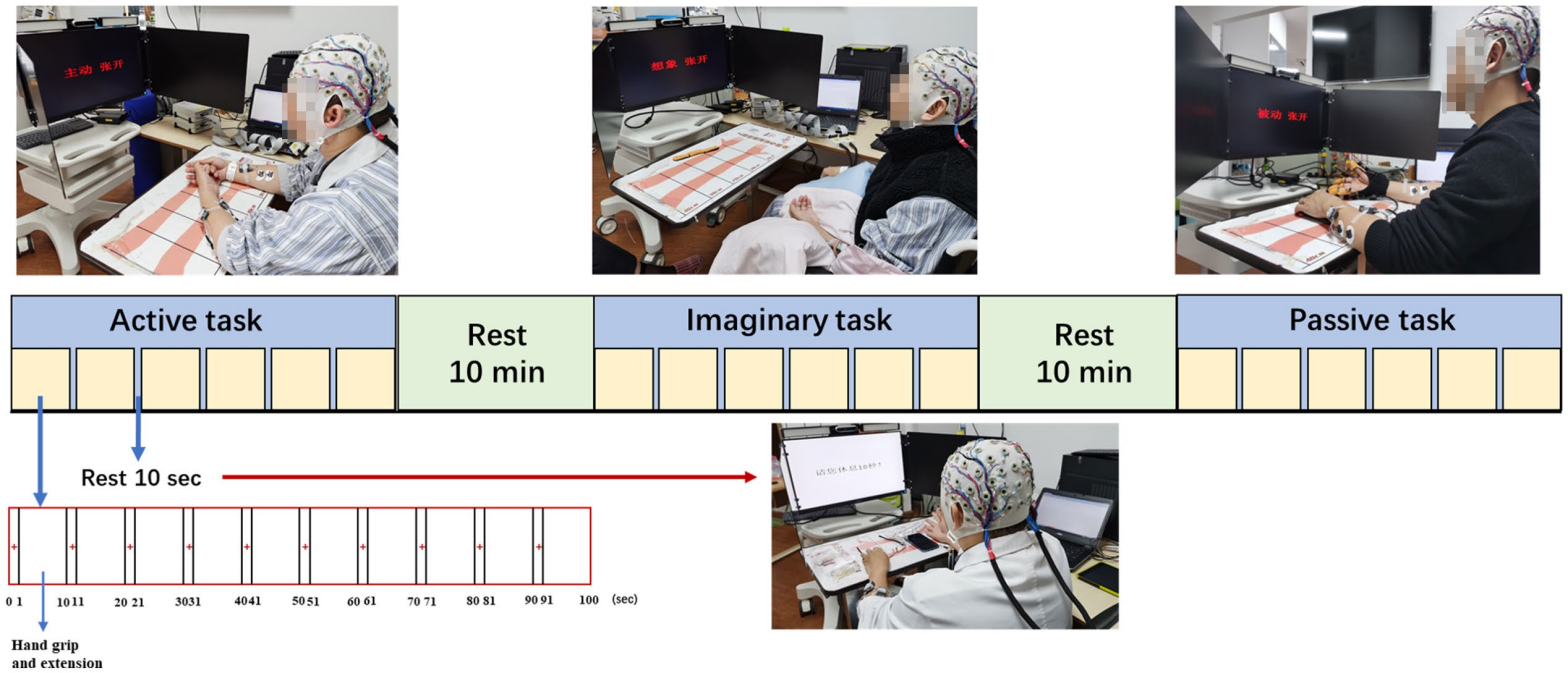
Study setup and experimental protocol.

Subjects were tested in a quiet room with sitting position and kept hands on the table for stability. There are three tasks (active, MI, and passive movement) performed in three sessions consisting of 60 trials each. Each trial started with a “+” in the center of the monitor for 1 s. After the “+” disappeared, text prompts corresponding to the task of 3 s hand grip and 6 s hand extension appeared. Ten seconds of rest between every 10 trials to avoid fatigue. Ten minutes of breaks between active, MI, and passive tasks to avoid interference with brain activity by different task types. In the active task, subjects were asked to grip and extend themselves with the affected hand. In the MI task, subjects were asked to imagine the affected hand grip and extension. In the passive task, robotic equipment (YS™ SY-HR06R Rehabilitation Robot Gloves, Shanghai, China) is worn on the affected hand and performs the movement. Robotic equipment helped all subjects grip and open their hands passively in a fixed time. The study design for active, MI and passive tasks is shown in Figure 1.

### 2.3. Clinical outcome measures

The Fugl-Meyer Assessment Upper Extremity subscale (FMA_UE) was used to assess motor impairment in the upper extremity. A higher score means less damage to the upper limb. The modified Ashworth Scale (MAS) and Manual Muscle Test (MMT) were used to assess the muscle tone and strength of the elbow and wrist flexor and extensor, respectively. A higher score indicated more severe spasticity and stronger muscles. Grip and pinch strength were used to measure muscle fitness and overall muscle strength. The modified Barthel Index (MBI) is an 11-item scale that assesses the activity of daily living capacity. The Berg Balance Scale (BBS) was used to measure balance and control levels.

### 2.4. EMG recordings

Four bipolar surface electromyograph electrodes (Noraxon U.S.A. INC., Clinical DTS) were fixed to flexor digitorum superficialis and extensor digitorum on bilateral upper extremities to monitor movement and ensure the absence of EMG activity in the relevant muscle groups during MI and passive task.

### 2.5. EEG recordings and preprocessing

EEG signals were obtained with a 64 Ag/AgCl scalp electrode positioned according to the international 10–20 system at a sampling rate of 1,000 Hz (Brain Products, Gilching, Germany). EEG data was exported to Python 3.8 and PyCharm 2022.1.3 (Community Edition) for subsequent preprocessing and analysis. The bandpass filter range was 1–60 Hz and the sampling rate was down to 500 Hz. A 50 Hz notch filter and independent component analysis (ICA) were adopted to mitigate the effect of power line noise and eye movement artifacts in pre-processing. EEG signals were filtered into eight frequency bands: delta (1–4 Hz), low alpha (8–10 Hz), high alpha (10–13 Hz), low beta (13–20 Hz), high beta (20–30 Hz) and gamma (30–48 Hz). Then, we flipped the scalp electrode position data of subjects with right-sided lesions along the mid-sagittal plane to perform a group analysis of all 15 subjects.

### 2.6. Task-related coherence (TRCoh)

For normalization of the underlying distribution, coherence estimate was subjected to a hyperbolic inverse tangent (tanh^−1^) transformation. Then, to reduce inter-subject variability, coherence estimates recorded during task execution were normalized with coherence estimates during rest (27). TRCoh was derived using the following formula:

$$TRCoh=\tanh^{-1}\left(Coh_{task}\right)-\tanh^{-1}\left(Coh_{rest}\right)$$

Coherence is a measure of the linear association between two signals and is calculated by the square of the absolute value of the coherence function (K), where K is the cross-spectral density, according to the formula (28):

$$COH_{xy}\left(f\right)={\left|K_{xy}\left(f\right)\right|}^{2}=\frac{{\left|S_{xy}\left(f\right)\right|}^{2}}{S_{xx}\left(f\right)S_{yy}\left(f\right)},$$

where S denotes the cross-spectrum at any given frequency, and x and y are regions, $S_{xy}\left(f\right)$ , $S_{xx}\left(f\right)$ , and $S_{yy}\left(f\right)$ are power spectral densities between x(t) and y(t) , and, respectively. $COH_{xy}\left(f\right)$ is a bounded measure taking values from 0 to 1, where 0 indicates that there is no linear association between x(t) and y(t) at frequency f, and 1 indicates a perfect linear association between x(t) and y(t) at frequency f.

### 2.7. Network topology

Three different weighted network indices were evaluated in this study based on graph theory (29): (1) nodal clustering coefficient (CC), (2) characteristic path length (CPL), and (3) small-world (SW).

A coherence matrix was used identically as a graph (G) consisting of node (N) and edge (E), and coherence presents the weight (w) of an edge between two nodes.

CC indicates how well a brain region is clustered with neighboring regions (segregation), quantifying by the degree of clustering among three nodes, creating a triangle. To compute nodal CC, the values of neighboring triangles of the node should be computed before:

$$t_{i}=\frac{1}{2}\underset{j,h\in N}{\sum }\left(w_{ij}\times w_{jh}\times w_{hi}\right),$$

where N represents all nodes included in G, and j and h are all possible pairs of adjacent nodes that create triangles with a specific node. Nodal CC is defined as:

$$C_{i}=\frac{1}{n}\underset{i\in N}{\sum }\frac{2t_{i}}{k_{i}\left(k_{i}-1\right)},$$

Where n is the number of nodes, and $k_{i}$ is the number of all connected nodes for a specific node. In this study, n and $k_{i}$ were 63 and 62, respectively, because the number of nodes was 63 and we assumed that each node was fully connected to the other nodes.

CPL (L) represents the overall connectivity of the entire network structure, and is defined as:

$$L=\frac{1}{n\left(n-1\right)}\underset{i,j\in N,i\neq j}{\sum }d_{ij},$$

where $d_{ij}$ indicates the shortest distance between two nodes ( i and j ), quantified by an inverse of the weight, when using a fully connected weight graph.

SW indicates how brain networks work cost effectively when transferring information from one region to another. SW is defined as:

$$S=\frac{CC/CC_{r}}{PL/PL_{r}}$$

where $CC_{r}$ and $PL_{r}$ represent CC and CPL equivalent random networks with the same degree distribution.

### 2.8. Select ROIs for brain connectivity and network properties

**Figure 2:**
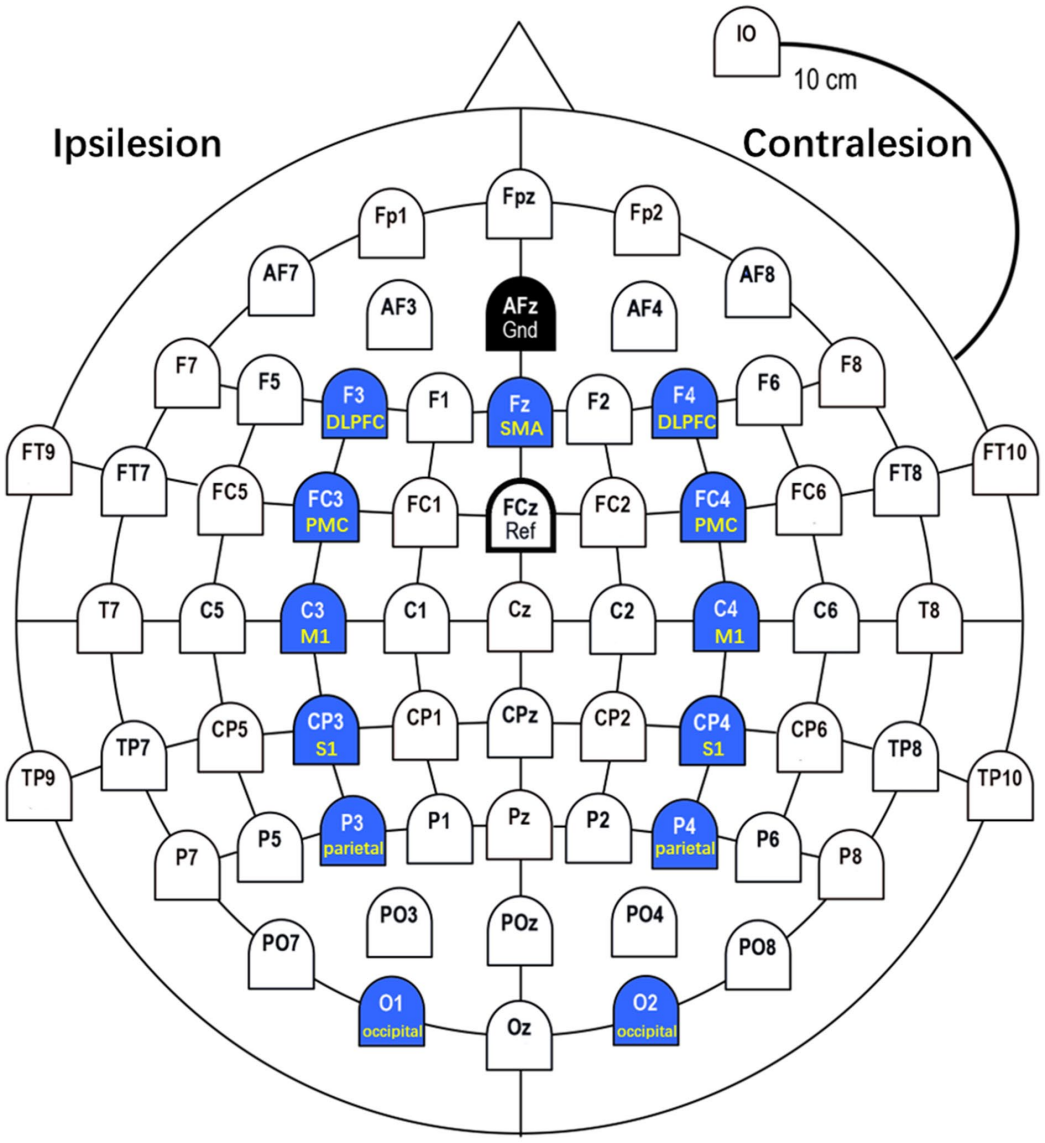
ROIs (Regions of Interest). In this study, we selected 13 ROIs in M1 (C3, C4), PMC (FC3, FC4), SMA (Fz), S1 (CP3, CP4), DLPFC (F3, F4), parietal (P3, P4), and occipital (O1, O2) based on previous studies on motor tasks.

In this study, we selected 13 ROIs (C3, C4, FC3, FC4, Fz, CP3, CP4, F3, F4, P3, P4, O1, O2) in primary motor cortices (M1), premotor cortices (PMC), supplementary motor area (SMA), somatosensory cortices (S1), dorsolateral prefrontal cortex (DLPFC), parietal areas and occipital areas based on previous studies on motor tasks. Kim et al. showed meaningful features in brain networks between M1, S1, SMA, and PMC during active motor and MI task (24). Lam et al. asserted that connectivity within and between motor (M1, SMA) and frontoparietal (DLPFC) networks correlates with motor outcome after stroke (30). Van Wijk et al. identified that the interaction between occipital areas and primary motor cortices led to a greater gamma increase and beta decrease in occipital areas during motor imagery (31). Based on these studies, we analyzed brain connectivity and network properties around the M1, PMC, SMA, S1, DLPFC, parietal, and occipital regions during three motor tasks. The ROIs is shown in Figure 2.

### 2.9. Statistical analyses

Statistical analyses were performed with SPSS version 25.0 (SPSS Inc., Chicago, IL, United States) and figures were drawn with Python 3.8 and PyCharm 2022.1.3 (Community Edition). The Mann–Whitney U test was used to analyze the difference in clinical outcome measures between subacute and chronic subjects, including FMA_UE, Grip and pinch strength, MBI, and BBS. (Chi-square was used to compare the clinical outcome measures of MAS) and MMT between two groups. Two-way repeated ANOVAs taking task (3 levels: active, MI and passive task) as the within-subject factor and group as the between-subject factor were performed on the strength of connectivity, and network parameters (nodal CC, CPL, and S). Bonferroni correction was used to adjust *p* values for multiple tests of functional connectivity and network parameters between different tasks. Results are presented as mean with standard deviations (SD). The statistical significance was set at *p* < 0.05 with a 2-sided test.

## 3. Result

### 3.1. Clinical outcome measures between two groups

Using Mann–Whitney U test and Chi-square, there were no significant differences in each clinical outcome measure between subacute and chronic subjects.

### 3.2. Significant frequency band and connectivity

**Figure 3:**
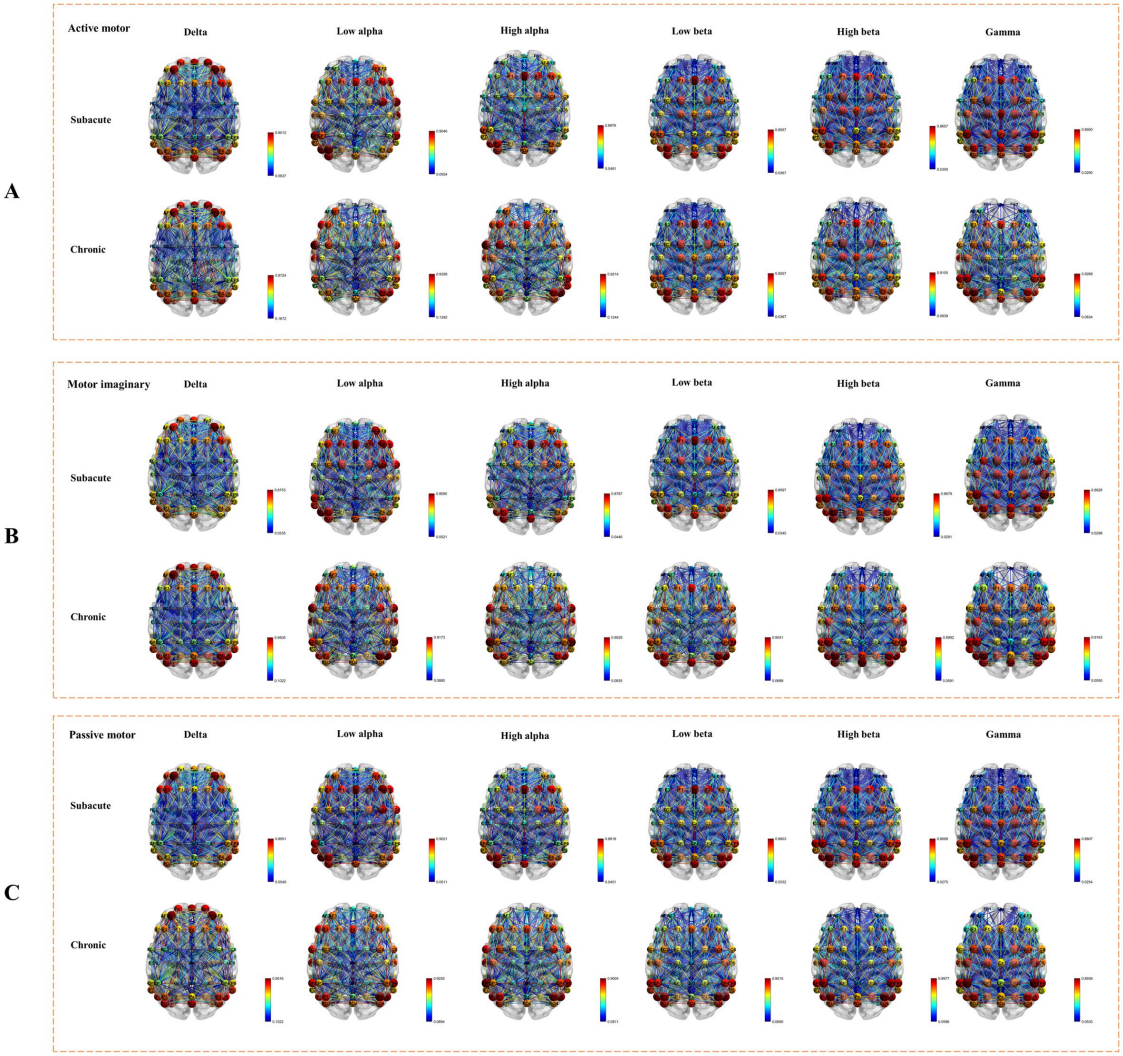
Whole brain connectivity under active, MI, and passive conditions. **(A)** The connectivity under active task condition. **(B)** The connectivity under MI task condition. **(C)** The connectivity under passive task condition.

Using the TRCoh approach, we calculated delta, low alpha, high alpha, low beta, high beta, and gamma band coherence for each condition, followed by significant tests. Whole brain connectivity under active, MI, and passive conditions was shown in Figure 3. For each significant ROIs connection, the mean, standard deviation, and value of p of these functional connectivity measures (using two-way repeated ANOVAs) in different frequency bands are shown in Tables 2–7 respectively.

- Significant connectivity in the delta band

**Table 2:** Significance connectivity in delta band (1–4 Hz).

connection	Task	SG	CG	*p*-Value _task×group_	*p*-Value _task_	*p*-Value _group_
		Mean (SD)	Mean (SD)			
C3-CP3	active task	0.80 (0.10)	0.84 (0.05)	0.792	0.031*	0.741
	MI task	0.68 (0.17)	0.76 (0.19)			
	passive task	0.75 (0.10)	0.75 (0.17)			
C4-F4	active task	0.27 (0.09)	0.40 (0.13)	0.029*	0.601	0.788
	MI task	0.44 (0.30)	0.32 (0.17)			
	passive task	0.41 (0.18)	0.51 (0.13)			
C4-P3	active task	0.18 (0.04)	0.34 (0.12)	0.003*	0.703	0.574
	MI task	0.34 (0.13)	0.30 (0.14)			
	passive task	0.36 (0.10)	0.30 (0.12)			
C4-P4	active task	0.55 (0.19)	0.68 (0.10)	0.022*	0.735	0.630
	MI task	0.63 (0.05)	0.48 (0.23)			
	passive task	0.66 (0.05)	0.53 (0.15)			
F3-CP3	active task	0.30 (0.14)	0.38 (0.22)	0.025*	0.753	0.656
	MI task	0.28 (0.11)	0.26 (0.07)			
	passive task	0.35 (0.17)	0.19 (0.04)			
F4-FC4	active task	0.73 (0.02)	0.64 (0.21)	0.041*	0.703	0.826
	MI task	0.73 (0.17)	0.75 (0.11)			
	passive task	0.66 (0.19)	0.81 (0.10)			
O1-O2	active task	0.86 (0.03)	0.91 (0.03)	0.013*	0.719	0.151
	MI task	0.82 (0.10)	0.90 (0.06)			
	passive task	0.84 (0.07)	0.87 (0.05)			

SG, subacute group; CG, chronic group; SD, standard deviation. *p < 0.05, ***p < 0.001.

**Table 3:** Significant connectivity in low alpha band (8–10 Hz).

connection	Task	SG	CG	*p*-Value _task×group_	*p*-Value _task_	*p*-Value _group_
		Mean (SD)	Mean (SD)			
C3-CP3	active task	0.83 (0.02)	0.83 (0.04)	0.850	0.021*	0.886
	MI task	0.81 (0.04)	0.82 (0.04)			
	passive task	0.82 (0.01)	0.82 (0.04)			
C3-P4	active task	0.43 (0.02)	0.55 (0.06)	0.933	1.000	0.015*
	MI task	0.43 (0.08)	0.55 (0.02)			
	passive task	0.43 (0.05)	0.55 (0.05)			
F3-P4	active task	0.57 (0.01)	0.69 (0.04)	0.506	0.964	0.042*
	MI task	0.59 (0.07)	0.66 (0.08)			
	passive task	0.58 (0.04)	0.67 (0.07)			
P3-P4	active task	0.14 (0.01)	0.17 (0.06)	0.033*	0.922	0.919
	MI task	0.16 (0.03)	0.14 (0.03)			
	passive task	0.15 (0.02)	0.15 (0.03)			
P4-CP3	active task	0.21 (0.03)	0.33 (0.07)	0.034*	0.971	0.160
	MI task	0.23 (0.03)	0.31 (0.08)			
	passive task	0.20 (0.04)	0.34 (0.09)			

SG, subacute group; CG, chronic group; SD, standard deviation. *p < 0.05, ***p < 0.001.

**Table 4:** Significance connectivity in the high alpha band (10–13 Hz).

connection	Task	SG	CG	*p*-Value _task×group_	*p*-Value _task_	*p*-Value _group_
		Mean (SD)	Mean (SD)			
C4-CP3	active task	0.35 (0.08)	0.56 (0.07)	0.293	0.916	0.008*
	MI task	0.35 (0.15)	0.55 (0.10)			
	passive task	0.40 (0.07)	0.48 (0.14)			
F3-C4	active task	0.26 (0.02)	0.35 (0.09)	0.059	0.028*	0.126
	MI task	0.27 (0.06)	0.41 (0.13)			
	passive task	0.19 (0.02)	0.29 (0.10)			
F3-CP4	active task	0.31 (0.10)	0.52 (0.06)	0.684	0.005*	0.085
	MI task	0.34 (0.06)	0.58 (0.06)			
	passive task	0.30 (0.06)	0.51 (0.04)			
FC3-CP4	active task	0.24 (0.09)	0.53 (0.08)	0.486	0.158	0.025*
	MI task	0.25 (0.08)	0.60 (0.08)			
	passive task	0.25 (0.04)	0.53 (0.04)			
FC4-CP3	active task	0.35 (0.12)	0.54 (0.09)	0.250	0.656	0.004*
	MI task	0.35 (0.15)	0.57 (0.10)			
	passive task	0.39 (0.09)	0.50 (0.11)			
Fz-CP4	active task	0.18 (0.04)	0.28 (0.10)	0.996	0.008*	0.165
	MI task	0.23 (0.04)	0.34 (0.08)			
	passive task	0.16 (0.01)	0.27 (0.08)			
O2-CP4	active task	0.20 (0.03)	0.27 (0.06)	0.799	0.001*	0.107
	MI task	0.25 (0.03)	0.32 (0.02)			
	passive task	0.18 (0.03)	0.27 (0.06)			
P3-CP3	active task	0.81 (0.05)	0.76 (0.06)	0.109	0.860	0.034*
	MI task	0.80 (0.05)	0.79 (0.04)			
	passive task	0.81 (0.05)	0.75 (0.07)			

SG, subacute group; CG, chronic group; SD, standard deviation. *p < 0.05, ***p < 0.001.

**Table 5:** Significant connectivity in low beta band (13–20 Hz).

connection	Task	SG	CG	*p*-Value _task×group_	*p*-Value _task_	*p*-Value _group_
		Mean (SD)	Mean (SD)			
C3-C4	active task	0.19 (0.02)	0.43 (0.08)	0.976	0.978	0.032*
	MI task	0.18 (0.06)	0.42 (0.09)			
	passive task	0.18 (0.02)	0.43 (0.18)			
C3-CP4	active task	0.22 (0.05)	0.46 (0.07)	0.724	0.587	0.024*
	MI task	0.19 (0.06)	0.47 (0.04)			
	passive task	0.21 (0.03)	0.51 (0.11)			
C3-FC4	active task	0.14 (0.03)	0.35 (0.08)	0.624	0.949	0.039*
	MI task	0.18 (0.05)	0.34 (0.12)			
	passive task	0.14 (0.03)	0.37 (0.15)			
C3-O2	active task	0.28 (0.01)	0.36 (0.04)	0.984	0.381	0.036*
	MI task	0.27 (0.07)	0.36 (0.02)			
	passive task	0.25 (0.03)	0.34 (0.07)			
C3-P4	active task	0.25 (0.05)	0.52 (0.07)	0.976	0.081	0.003*
	MI task	0.22 (0.08)	0.50 (0.01)			
	passive task	0.26 (0.04)	0.53 (0.08)			
C4-P3	active task	0.23 (0.02)	0.52 (0.08)	0.316	0.907	0.034*
	MI task	0.24 (0.08)	0.48 (0.07)			
	passive task	0.30 (0.05)	0.45 (0.22)			
F3-CP3	active task	0.16 (0.03)	0.36 (0.08)	0.160	0.064	0.030*
	MI task	0.14 (0.01)	0.23 (0.08)			
	passive task	0.15 (0.07)	0.27 (0.04)			
F3-P3	active task	0.18 (0.05)	0.14 (0.01)	0.883	0.021*	0.559
	MI task	0.21 (0.09)	0.19 (0.05)			
	passive task	0.15 (0.02)	0.13 (0.02)			
FC3-CP4	active task	0.17 (0.02)	0.46 (0.11)	0.884	0.652	0.021*
	MI task	0.20 (0.03)	0.48 (0.07)			
	passive task	0.16 (0.02)	0.47 (0.10)			
FC4-CP3	active task	0.18 (0.01)	0.49 (0.07)	0.609	0.982	0.017*
	MI task	0.22 (0.09)	0.46 (0.07)			
	passive task	0.22 (0.06)	0.47 (0.17)			
O1-CP3	active task	0.15 (0.01)	0.22 (0.07)	0.044*	0.592	0.182
	MI task	0.17 (0.04)	0.19 (0.02)			
	passive task	0.14 (0.03)	0.25 (0.07)			
P3-FC4	active task	0.28 (0.06)	0.58 (0.08)	0.356	0.942	0.003*
	MI task	0.31 (0.17)	0.57 (0.07)			
	passive task	0.34 (0.12)	0.52 (0.16)			
P4-CP3	active task	0.21 (0.06)	0.41 (0.08)	0.703	0.217	0.034*
	MI task	0.18 (0.06)	0.33 (0.05)			
	passive task	0.22 (0.06)	0.39 (0.10)			
P4-CP4	active task	0.76 (0.05)	0.71 (0.09)	0.016*	0.312	0.771
	MI task	0.76 (0.04)	0.73 (0.10)			
	passive task	0.76 (0.05)	0.77 (0.05)			
P4-FC3	active task	0.24 (0.04)	0.56 (0.03)	0.939	0.854	0.003*
	MI task	0.24 (0.10)	0.57 (0.03)			
	passive task	0.25 (0.07)	0.56 (0.07)			

SG, subacute group; CG, chronic group; SD, standard deviation. *p < 0.05, ***p < 0.001.

**Table 6:** Significant connectivity in high beta band (20–30 Hz).

connection	Task	SG	CG	*p*-Value _task×group_	*p*-Value _task_	*p*-Value _group_
		Mean (SD)	Mean (SD)			
C3-CP4	active task	0.19 (0.05)	0.43 (0.10)	0.888	0.523	0.043*
	MI task	0.17 (0.02)	0.40 (0.09)			
	passive task	0.21 (0.07)	0.46 (0.10)			
C3-P4	active task	0.18 (0.03)	0.46 (0.10)	0.887	0.356	0.003*
	MI task	0.16 (0.01)	0.43 (0.003)			
	passive task	0.22 (0.09)	0.47 (0.06)			
C4-CP3	active task	0.18 (0.05)	0.49 (0.10)	0.020*	0.688	0.090
	MI task	0.17 (0.03)	0.39 (0.12)			
	passive task	0.20 (0.07)	0.41 (0.19)			
C4-P3	active task	0.18 (0.03)	0.47 (0.06)	<0.001*	0.766	0.060
	MI task	0.18 (0.01)	0.39 (0.10)			
	passive task	0.24 (0.09)	0.38 (0.19)			
F3-C3	active task	0.27 (0.14)	0.52 (0.05)	0.614	0.097	0.047*
	MI task	0.21 (0.16)	0.50 (0.02)			
	passive task	0.28 (0.12)	0.53 (0.05)			
F3-O1	active task	0.21 (0.10)	0.32 (0.13)	0.228	0.031*	0.377
	MI task	0.20 (0.10)	0.32 (0.13)			
	passive task	0.17 (0.08)	0.25 (0.11)			
FC3-CP4	active task	0.18 (0.05)	0.41 (0.10)	0.916	0.547	0.044*
	MI task	0.17 (0.04)	0.42 (0.13)			
	passive task	0.21 (0.01)	0.44 (0.12)			
FC4-CP3	active task	0.15 (0.01)	0.48 (0.10)	0.070	0.691	0.047*
	MI task	0.16 (0.04)	0.40 (0.13)			
	passive task	0.18 (0.04)	0.43 (0.13)			
O1-Fz	active task	0.25 (0.19)	0.46 (0.08)	0.043*	0.601	0.319
	MI task	0.26 (0.19)	0.42 (0.06)			
	passive task	0.26 (0.15)	0.42 (0.08)			
P3-FC4	active task	0.17 (0.001)	0.53 (0.07)	0.020*	0.862	0.011*
	MI task	0.20 (0.07)	0.47 (0.08)			
	passive task	0.25 (0.07)	0.45 (0.12)			
P3-Fz	active task	0.22 (0.05)	0.38 (0.07)	0.693	0.599	0.030*
	MI task	0.20 (0.09)	0.33 (0.06)			
	passive task	0.19 (0.05)	0.32 (0.05)			
P3-O1	active task	0.27 (0.13)	0.69 (0.16)	0.403	0.138	0.011*
	MI task	0.33 (0.16)	0.63 (0.12)			
	passive task	0.25 (0.18)	0.60 (0.05)			
P4-FC3	active task	0.19 (0.03)	0.49 (0.03)	0.028*	0.544	0.003*
	MI task	0.15 (0.04)	0.52 (0.03)			
	passive task	0.21 (0.07)	0.50 (0.06)			

SG, subacute group; CG, chronic group; SD, standard deviation. *p < 0.05, ***p < 0.001.

**Table 7:** Significant connectivity in gamma band (30–48 Hz).

connection	Task	SG	CG	*p*-Value _task×group_	*p*-Value _task_	*p*-Value _group_
		Mean (SD)	Mean (SD)			
F4-P3	active task	0.18 (0.03)	0.37 (0.11)	0.380	0.587	0.045*
	MI task	0.20 (0.06)	0.36 (0.07)			
	passive task	0.19 (0.06)	0.33 (0.11)			
Fz-CP4	active task	0.23 (0.09)	0.19 (0.05)	0.019*	0.310	0.108
	MI task	0.23 (0.03)	0.14 (0.01)			
	passive task	0.14 (0.02)	0.17 (0.02)			
O1-CP3	active task	0.18 (0.04)	0.16 (0.02)	0.003*	0.718	0.810
	MI task	0.16 (0.02)	0.20 (0.06)			
	passive task	0.17 (0.02)	0.17 (0.04)			
O1-FC3	active task	0.15 (0.04)	0.27 (0.09)	0.347	0.356	0.019*
	MI task	0.15 (0.03)	0.30 (0.04)			
	passive task	0.14 (0.02)	0.23 (0.04)			
O1-FC4	active task	0.16 (0.05)	0.35 (0.11)	0.336	0.430	0.035*
	MI task	0.19 (0.04)	0.38 (0.10)			
	passive task	0.19 (0.11)	0.29 (0.11)			
O2-FC3	active task	0.17 (0.04)	0.35 (0.13)	0.699	0.714	0.007*
	MI task	0.15 (0.03)	0.34 (0.03)			
	passive task	0.16 (0.04)	0.30 (0.04)			
P4-CP4	active task	0.81 (0.02)	0.65 (0.02)	0.046*	0.668	0.081
	MI task	0.79 (0.04)	0.67 (0.04)			
	passive task	0.77 (0.04)	0.74 (0.08)			
P4-FC3	active task	0.17 (0.04)	0.47 (0.13)	0.464	0.550	0.026*
	MI task	0.18 (0.07)	0.42 (0.04)			
	passive task	0.18 (0.06)	0.39 (0.14)			
P4-O2	active task	0.33 (0.05)	0.58 (0.18)	0.017*	0.762	0.183
	MI task	0.32 (0.13)	0.59 (0.09)			
	passive task	0.38 (0.12)	0.43 (0.05)			

SG, subacute group; CG, chronic group; SD, standard deviation. *p < 0.05, ***p < 0.001.

There were 7 pairs of significant connections in the delta band. Significant task differences were found between ipsilesional M1 and S1 (C3-CP3, *F* = 9.422, *p* = 0.031). However, *post hoc* analyses (Bonferroni adjusted) indicated that the pairwise comparison of three tasks was not significant. Significant task × group interactions were observed on the connections between contralesional M1 and DLPFC (C4-F4, *F* = 9.835, *p* = 0.029), between contralesional M1 and ipsilesional parietal areas (C4-P3, *F* = 36.871, *p* = 0.003), between contralesional M1 and parietal areas (C4-P4, *F* = 11.538, *p* = 0.022), between ipsilesional DLPFC and S1 (F3-CP3, *F* = 10.555, *p* = 0.025), between contralesional DLPFC and PMC (F4-FC4, *F* = 7.839, *p* = 0.041), and between ipsilesional and contralesional occipital area (O1-O2, *F* = 15.811, *p* = 0.013).

- Significant connectivity in the low alpha band

There were 5 pairs of significant connections in the low alpha band. Significant task differences were found between ipsilesional M1 and S1 (C3-CP3, *F* = 11.915, *p* = 0.021). *Post hoc* analyses (Bonferroni adjusted) indicated that the C3-CP3 connection was comparable between active and MI tasks (*p* = 0.008). Significant group differences were found in the connections between ipsilesional M1 and contralesional parietal areas (C3-P4, *F* = 65.848, *p* = 0.015), and between ipsilesional DLPFC and contralesional parietal areas (F3-P4, *F* = 22.046, *p* = 0.042). Significant task × group interactions were observed on the connections between ipsilesional and contralesional parietal areas (P3-P4, *F* = 9.063, *p* = 0.033), and between contralesional parietal areas and ipsilesional S1 (P4-CP3, *F* = 8.838, *p* = 0.034).

- Significant connectivity in the high alpha band

There were 8 pairs of significant connections in the high alpha band. Significant task differences were observed between ipsilesional DLPFC and contralesional M1 (F3-C4, *F* = 9.990, *p* = 0.028), between ipsilesional DLPFC and contralesional S1 (F3-CP4, *F* = 26.265, *p* = 0.005), between SMA and contralesional S1 (Fz-CP4, *F* = 20.676, *p* = 0.008), between contralesional occipital area and S1 (O2-CP4, *F* = 59.219, *p* = 0.001). *Post hoc* analyses (Bonferroni adjusted) indicated that the F3-CP4 connection was comparable between MI and passive task. And the O2-CP4 connection was also comparable between active and MI task, as well as MI and passive task (*p* = 0.029 and *p* = 0.034, respectively). Group differences were significant in the connections between contralesional M1 and ipsilesional S1 (C4-CP3, *F* = 123.177, p = 0.008), between ipsilesional PMC and contralesional S1 (FC3-CP4, *F* = 39.213, *p* = 0.025), between contralesional PMC and ipsilesional S1 (FC4-CP3, *F* = 254.819, *p* = 0.004), and between ipsilesional parietal areas and S1 (P3-CP3, *F* = 27.723, p = 0.034).

- Significant connectivity in the low beta band

There were 15 pairs of significant connections in the low beta band. Significant task × group interactions were found on the connections between ipsilesional occipital area and S1 (O1-CP3, *F* = 7.513, *p* = 0.044), and between contralesional parietal areas and S1 (P4-CP4, *F* = 13.631, *p* = 0.016). Task differences were significant in the connection between ipsilesional DLPFC and parietal areas (F3-P3, *F* = 11.693, *p* = 0.021). However, *post hoc* analyses (Bonferroni adjusted) indicated that the pairwise comparison of three tasks was not significant. The strength of connectivity in SG were all significantly lower than the CG on the connections between bilateral M1 (C3-C4, *F* = 29.400, *p* = 0.032), between ipsilesional M1 and contralesional S1 (C3-CP4, *F* = 40.086, *p* = 0.024), between ipsilesional M1 and contralesional PMC (C3-FC4, *F* = 24.137, *p* = 0.039), between ipsilesional M1 and contralesional occipital area (C3-O2, *F* = 26.093, *p* = 0.036), between ipsilesional M1 and contralesional parietal areas (C3-P4, *F* = 294.146, *p* = 0.003), between contralesional M1 and ipsilesional parietal areas (C4-P3, *F* = 27.678, *p* = 0.034), between ipsilesional DLPFC and S1 (F3-CP3, *F* = 31.897, *p* = 0.030), between ipsilesional PMC and contralesional S1 (FC3-CP4, *F* = 46.568, *p* = 0.021), between ipsilesional S1 and contralesional PMC (FC4-CP3, *F* = 56.512, *p* = 0.017), between ipsilesional parietal areas and contralesional PMC (P3-FC4, *F* = 360.911, *p* = 0.003), between contralesional parietal areas and ipsilesional S1 (P4-CP3, *F* = 28.072, *p* = 0.034), and between contralesional parietal areas and ipsilesional PMC (P4-FC3, *F* = 359.422, *p* = 0.003), respectively.

- Significant connectivity in the high beta band

There were 13 pairs of significant connections in the high beta band. Significant task × group interactions were observed on the connections between contralesional M1 and ipsilesional S1 (C4-CP3, *F* = 12.278, *p* = 0.020), between contralesional M1 and ipsilesional parietal areas (C4-P3, *F* = 178.855, *p* < 0.001), between ipsilesional occipital area and SMA (O1-Fz, *F* = 7.604, *p* = 0.043). Task differences were significant in the connection between (F3-O1, *F* = 9.443, *p* = 0.031). However, *post hoc* analyses (Bonferroni adjusted) showed that the pairwise comparison of three tasks was not significant. The strength of connectivity in SG was significantly lower than CG on the connections between ipsilesional M1 and contralesional S1 (C3-CP4, *F* = 22.027, p = 0.043), between ipsilesional M1 and contralesional parietal areas (C3-P4, *F* = 349.550, *p* = 0.003), between ipsilesional DLPFC and M1 (F3-C3, *F* = 19.705, *p* = 0.047), between ipsilesional PMC and contralesional S1 (FC3-CP4, *F* = 21.012, *p* = 0.044), between contralesional PMC and ipsilesional S1 (FC4-CP3, *F* = 19.913, p = 0.047), between ipsilesional parietal areas and SMA (P3-Fz, *F* = 31.587, *p* = 0.030), between ipsilesional parietal areas and ipsilesional occipital area (P3-O1, *F* = 91.371, *p* = 0.011). Significant task × group interactions and group difference significant were observed on the connections between ipsilesional parietal areas and contralesional PMC (P3-FC4, F _task×group_ = 12.025, P _task×group_ = 0.020, F _group_ = 93.588, P _group_ = 0.011), and between contralesional parietal areas and ipsilesional PMC (P4-FC3, F _task×group_ = 9.990, P _task×group_ = 0.028, F _group_ = 367.452, P _group_ = 0.003).

- Significant connectivity in the gamma band

There were 9 pairs of significant connections in the gamma band. Significant task × group interactions were found on the connections between SMA and contralesional S1 (Fz-CP4, *F* = 12.652, *p* = 0.019), between ipsilesional occipital area and S1 (O1-CP3, *F* = 37.129, *p* = 0.003), between contralesional parietal areas and S1 (P4-CP4, *F* = 7.312, *p* = 0.046), between contralesional parietal and occipital area (P4-O2, *F* = 13.130, *p* = 0.017). The connectivity strength in SG was significantly lower than CG on the connections between contralesional DLPFC and ipsilesional parietal areas (F4-P3, *F* = 20.821, *p* = 0.045), between ipsilesional occipital area and PMC (O1-FC3, *F* = 50.804, p = 0.019), between ipsilesional occipital area and contralesional PMC (O1-FC4, *F* = 27.368, *p* = 0.035), between contralesional occipital area and ipsilesional PMC (O2-FC3, *F* = 143.301, *p* = 0.007), between contralesional parietal areas and ipsilesional PMC (P4-FC3, *F* = 36.379, *p* = 0.026).

### 3.3. Characteristics of brain network properties in ROIs

In this study, we analyzed the characteristics of the nodal CC, CPL, and SW under active, MI, and passive motor task conditions.

#### 3.3.1. Nodal CC

**Figure 4:**
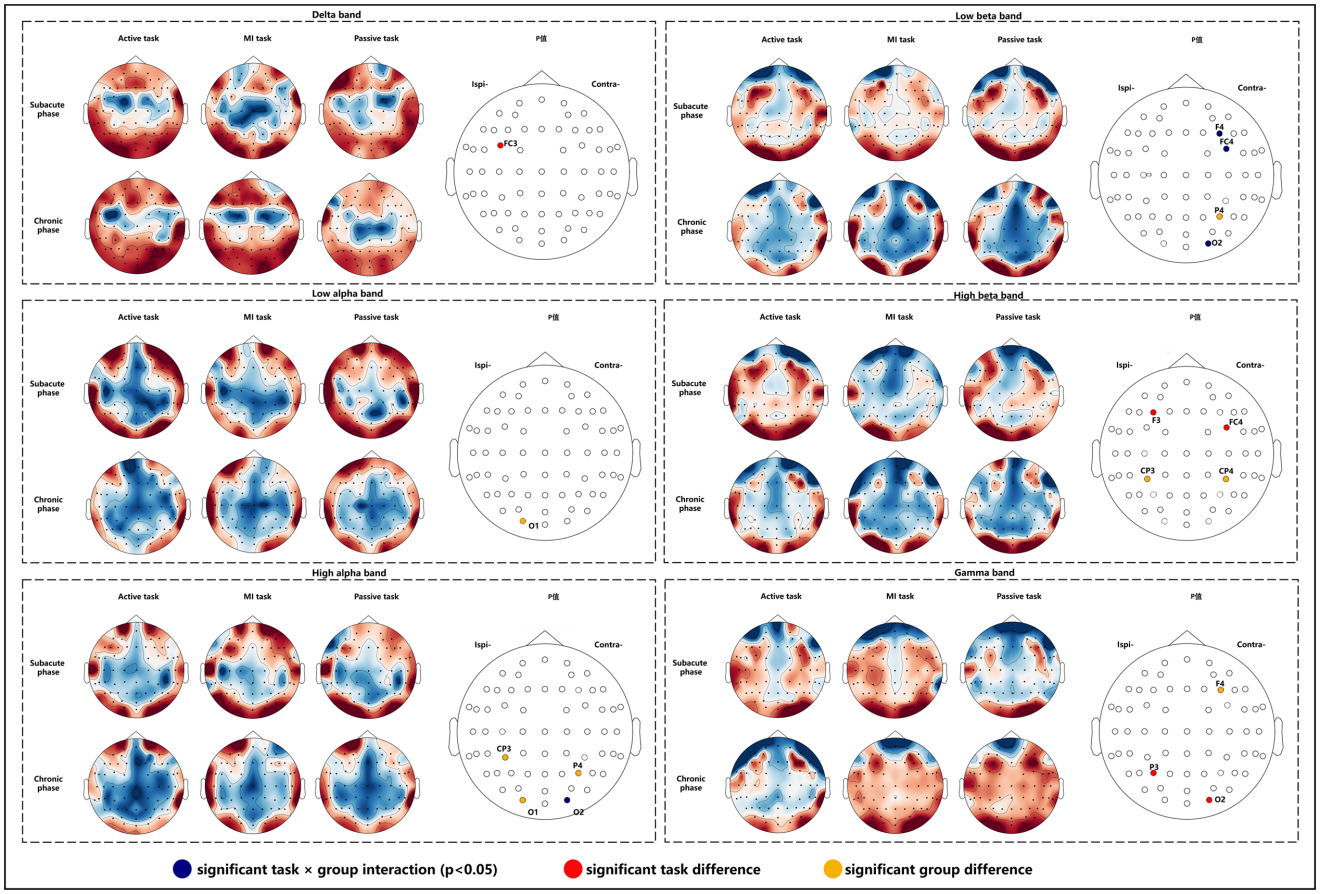
Nodal CC alterations in subacute and chronic stroke survivors.

We found that task × group interactions was significant mainly in the occipital area (*F* = 14.870, *p* = 0.014) in high alpha band, and in the contralesional DLPFC, PMC and occipital area (*F* = 19.566, *p* = 0.009; *F* = 26.009, *p* = 0.005; *F* = 23.997, *p* = 0.006) in low beta band. Significant task differences were observed in ipsilesional PMC (*F* = 9.824, *p* = 0.029) in delta band, ipsilesional DLPFC and contralesional PMC (*F* = 14.590, *p* = 0.015; *F* = 24.678, p = 0.006) in high beta band, and ipsilesional parietal area and contralesional occipital area (*F* = 22.213, *p* = 0.007; *F* = 10.854, *p* = 0.024) in gamma band. *Post hoc* analyses (Bonferroni adjusted) indicated that the higher value on FC3 of passive task compared to active task in delta band, the higher value on FC4 of active task compared to MI task in high beta band, and the higher value on P3 of MI task compared to passive task in gamma band. Significant group differences were found in the ipsilesional occipital area (*F* = 25.377, *p* = 0.037) in the low alpha band, the contralesional parietal area (*F* = 19.551, *p* = 0.048), ipsilesional S1 and occipital area (*F* = 41.076, *p* = 0.023; *F* = 44.431, *p* = 0.022) in the high alpha band, the contralesional parietal area (*F* = 24.017, *p* = 0.039) in the low beta band, bilateral S1 (*F* = 38.611, *p* = 0.025; *F* = 32.062, *p* = 0.030) in high beta band, and contralesional DLPFC (*F* = 39.967, *p* = 0.024) in gamma band (Figure 4).

#### 3.3.2. Characteristic PL

In low beta, high beta and gamma bands, CPL in subacute subjects was significantly lower than in chronic subjects (*F* = 21.294, *p* = 0.044; *F* = 107.349, *p* = 0.009; *F* = 161.922, *p* = 0.006) (Table 8).

**Table 8:** Characteristic path length (CPL) on each motor task.

Band	Task	SG	CG	*p*-Value _task×group_	*p*-Value _task_	*p*-Value _group_
		Mean (SD)	Mean (SD)			
Delta (1-4 Hz)	active task	0.23 (0.01)	0.24 (0.02)	0.261	0.765	0.248
	MI task	0.22 (0.02)	0.23 (0.02)			
	passive task	0.23 (0.01)	0.22 (0.01)			
Low alpha (8–10 Hz)	active task	0.21 (0.01)	0.21 (0.004)	0.877	0.416	0.845
	MI task	0.21 (0.03)	0.21 (0.01)			
	passive task	0.21 (0.02)	0.20 (0.01)			
High alpha (10–13 Hz)	active task	0.18 (0.02)	0.20 (0.01)	0.944	0.173	0.225
	MI task	0.18 (0.04)	0.20 (0.004)			
	passive task	0.18 (0.02)	0.20 (0.01)			
Low beta (13–20 Hz)	active task	0.14 (0.01)	0.18 (0.005)	0.167	0.807	0.044*
	MI task	0.15 (0.02)	0.18 (0.01)			
	passive task	0.14 (0.02)	0.18 (0.02)			
High beta (20–30 Hz)	active task	0.14 (0.01)	0.18 (0.01)	0.714	0.306	0.009*
	MI task	0.13 (0.01)	0.17 (0.01)			
	passive task	0.13 (0.02)	0.17 (0.01)			
Gamma (30–48 Hz)	active task	0.15 (0.01)	0.16 (0.01)	0.682	0.092	0.006*
	MI task	0.15 (0.01)	0.16 (0.002)			
	passive task	0.13 (0.01)	0.15 (0.01)			

SG, subacute group; CG, chronic group; SD, standard deviation. *p < 0.05, ***p < 0.001.

#### 3.3.3. Small-world

All stroke survivors tend to have small-world properties (SW > 1). However, there were no significant differences between subacute and chronic subjects (Table 9).

**Table 9:** Small-world (SW) on each motor task.

Band	Task	SG	CG	*p*-Value _task×group_	*p*-Value _task_	*p*-Value _group_
		Mean (SD)	Mean (SD)			
Delta (1-4 Hz)	active task	2.42 (0.08)	2.43 (0.08)	0.914	0.448	0.475
	MI task	2.39 (0.07)	2.44 (0.03)			
	passive task	2.37 (0.05)	2.41 (0.07)			
Low alpha (8–10 Hz)	active task	2.34 (0.06)	2.36 (0.03)	0.939	0.442	0.298
	MI task	2.36 (0.05)	2.38 (0.03)			
	passive task	2.34 (0.06)	2.36 (0.05)			
High alpha (10–13 Hz)	active task	2.31 (0.13)	2.45 (0.03)	0.967	0.134	0.205
	MI task	2.28 (0.13)	2.42 (0.02)			
	passive task	2.30 (0.12)	2.44 (0.01)			
Low beta (13–20 Hz)	active task	2.28 (0.12)	2.44 (0.12)	0.341	0.816	0.064
	MI task	2.35 (0.17)	2.37 (0.17)			
	passive task	2.24 (0.14)	2.41 (0.14)			
High beta (20–30 Hz)	active task	2.35 (0.17)	2.40 (0,01)	0.518	0.377	0.244
	MI task	2.27 (0.02)	2.44 (0.12)			
	passive task	2.35 (0.14)	2.50 (0.11)			
Gamma (30–48 Hz)	active task	2.48 (0.12)	2.42 (0.05)	0.440	0.098	0.929
	MI task	2.48 (0.11)	2.42 (0.04)			
	passive task	2.33 (0.11)	2.43 (0.08)			

SG, subacute group; CG, chronic group; SD, standard deviation. *p < 0.05, ***p < 0.001.

## 4. Discussion

The purpose of this pilot study was to investigate brain connectivity and network properties in subacute and chronic survivors during active, MI, and passive motor tasks. To find significant changes in coherences and brain network properties in three motor tasks, we performed two-way repeated ANOVA (*p* < 0.05) between coherences and network properties of ROIs on the frequency band that have been proven to be associated with motor function. As a result, the main effects of task and group as well as interaction show significant differences in coherence, nodal CC and CPL in delta (1–4 Hz), low alpha (8–10 Hz), high alpha (10–13 Hz), low beta (13–20 Hz), high beta (20–30 Hz), and gamma (30–48 Hz) bands.

In this study, clinical outcome measures were not significantly different between subacute and chronic survivors in statistical analysis. However, the differences are clinically significant in FMA_UE scores. Therefore, differences in FMA_UE scores for included patients should be limited to a certain range. Besides, the FMA_UL score was zero for three patients. It seems difficult for them to complete the active hand grip. In fact, the active task refers to the attempt to grasp the hand. In addition, there were ceiling and floor effects for FMA-UL when assessing upper limb motor impairment for stroke survivors. And they actually have slight hand mobility but not up to the FMA-UL standard. Hence, they were able to complete active tasks.

### 4.1. Functional connectivity between ROIs

Strens et al. indicated that changes in coherence likely reflected changes in the degree of coupling between regions (21). In this study, we calculated the TRCoh between motor-related areas to reflect changes in ROIs coupling between subacute and chronic survivors during motor tasks. Table 2–7 shows functional connectivity in delta, low alpha, high alpha, low beta, high beta, and gamma bands. Our results showed that significant interactions were found on quite a few connections linked to frontal, parietal and occipital lobes in each frequency band, particularly in the delta band. This represented the higher-order motor cortex, like the frontoparietal network (FPN), which was affected by the different stages of stroke recovery and task type. As far as we know, FPN has been demonstrated to be involved in movement planning and execution, and to provide corrective movement plans according to actual requirement (32). Recently, delta coherence has been a growing concern, which plays an important role when large-scale, distant cortical networks coordinate their neural activity, especially in the context of modulating attention or motivation (33). One interpretation of the apparent delta-band interaction is that the large-scale coordination of FPN in modulating attention and motivation was influenced by task and stroke stage, and further study is needed to better understand post-stroke motor recovery.

We also observed that the coupling strength of PMC, M1, and S1 and several connections linked to frontal, parietal, and occipital lobes in subacute subjects were lower than in chronic subjects in low alpha, high alpha, low beta, and high beta bands. These results showed that the coupling strength in the above areas was stronger for chronic patients than for subacute patients in the alpha and beta bands. Alpha and beta oscillatory processes are widely investigated in post-stroke motor impairment. The former was thought to reflect alternating cortical states of excitation and inhibition, and the latter was closely related to motor processes (34, 35). However, why is the coupling strength stronger in chronic patients than in subacute patients? One possible interpretation is that multiple activation zones for motor functional networks compensate for chronic stroke participants. That is, more resources need to be mobilized to carry out the same tasks. Previous studies have also supported the deduction of this result. De Vico et al. found that higher interhemispheric connectivity in the parieto-occipital region may result from greater attentional resource engagement for patients during motor imagery with affected hand (23). Strens et al. argued that increased task-related coupling between cortical areas may dynamically compensate for brain damage after stroke (21). It was also demonstrated that the mechanism of functional reorganization was different between subacute and chronic phases. Normally, the subacute phase is characterized by spontaneous neuroplastic changes, and the chronic phase is characterized by new patterns of neural activity established with spontaneous plasticity ended (34). Therefore, this possible mechanism might imply that compensatory strategies used in clinical practice are debatable.

In addition, significant task differences after Bonferroni correction were only shown in the low and high alpha bands. This finding showed that although several studies have demonstrated that active, MI, and passive tasks have similar cortical activations, the required cognitive load was not the same in different motor task (36, 37). Ogawa et al. have similar findings with higher directed functional connectivity from the contralateral dorsal PMC to M1 in ME than in MI (38). We demonstrated a higher coupling between ipsilesional M1 and S1 in active task than in MI task in low alpha band. And the coupling between ipsilesional DLPFC and contralesional S1 was higher in MI task than in passive task in high alpha band. It is likely that in the brain after stroke, higher cognitive demands in the active task were greater than in the MI task, and the MI task was greater than in the passive task.

### 4.2. Characteristics of a brain network

Our main network properties are the nodal CC, CPL, and SW. The nodal CC indicates how well a brain region is clustered with neighboring regions, and the higher value means the more important role in the local range. The higher CPL value represents the lower transfer efficiency of the entire network structure, and the small-world indicates how efficiently the brain network processes information (39). Normally, a healthy brain exhibited higher nodal CC and lower CPL, known as a small-world network model (22). After stroke, global functional integration was disrupted, information transmission efficiency decreased, and a shift to random networks was observed in rest-state brain networks (40, 41). In this study, nodal CC was found to increase in bilateral S1, ipsilesional occipital area, and contralesional DLPFC and parietal area for chronic subjects compared to subacute subjects in the alpha, beta, and gamma bands, as well as with CPL. These findings demonstrated that subacute patients were characterized by higher transfer efficiency of the entire brain network and weak local nodal effects. At the chronic stage, the transfer efficiency was weakened and the local nodal role was strengthened. The interpretation of these findings is that large-scale communication between regions was emphasized in the subacute stage with higher transfer efficiency, and in the chronic stage, spontaneous recovery gradually disappears with weakened large-scale brain communication and the emphasis on local nodal role. However, as we lack information on serial TRCoh values, we are unable to confirm this point with this study.

### 4.3. Limitations

Several limitations of this study should be noted. First, longitudinal changes in brain network connectivity and properties under task conditions from subacute to chronic were not identified due to cross-sectional research design. Second, the sample size is relatively small and the clinical characteristics of stroke patients may have some impact on the outcome. A larger sample size, a similar degree of functional impairment, consistent hemispheric lesion, and more stringent lesion site restrictions should be considered in our next study. Third, clinical outcome evaluations were not comprehensive, as the Action Research Arm Test (ARAT) and the National Institute of Health Stroke Scale (NIHSS) should be considered in our future study. Finally, the volume conduction problem of EEG makes it difficult to accurately localize the source activity. Therefore, combining MRI or fNIRS for source localization will be necessary in the future.

## 5. Conclusion

In conclusion, this study demonstrated the characteristics of brain coupling and network properties between subacute and chronic survivors after stroke. Subacute survivors were characterized by higher transfer efficiency of the entire brain network and weak local nodal effects. Transfer efficiency was reduced, the local nodal role was strengthened, and more neural resources needed to be mobilized to perform motor tasks for chronic survivors. Overall, these results may help to understand the remodeling pattern of the brain network for different post-stroke stages on task conditions and the mechanism of spontaneous recovery.

## Data availability statement

The original contributions presented in the study are included in the article, further inquiries can be directed to the corresponding author.

## Ethics statement

The studies involving human participants were reviewed and approved by the Institutional Review Boards of Huashan Hospital. The patients/participants provided their written informed consent to participate in this study.

## Author contributions

JJ design the study. YL and ZJ performed the experiment. YL, GZ, HS, and XK analyzed the data. YL wrote the manuscript. All authors contributed to the article and approved the submitted version.

## Funding

This work was supported by the National Natural Science Foundation of China (Grant number 91948302, 82202798, and 82021002), the National Key Research and Development Program of the Ministry of Science and Technology of the People’s Republic of China (Grant numbers 2018YFC2002300 and 2018YFC2002301), the Shanghai Sailing Program (22YF1404200), and the Shanghai Jing‘an District Key discipline construction of health system Program (no 2021ZB02).

## Conflict of interest

The authors declare that the research was conducted in the absence of any commercial or financial relationships that could be construed as a potential conflict of interest.

## Publisher’s note

All claims expressed in this article are solely those of the authors and do not necessarily represent those of their affiliated organizations, or those of the publisher, the editors and the reviewers. Any product that may be evaluated in this article, or claim that may be made by its manufacturer, is not guaranteed or endorsed by the publisher.

## References

[1] Tu WJ Chao B Wang L. Prevalence of stroke in China: overestimated? Lancet Public Health. (2022) 7:e404. doi: 10.1016/S2468-2667(22)00066-4 35487227

[2] Winstein CJ Stein J Arena R Bates B Cherney LR Cramer SC. Guidelines for adult stroke rehabilitation and recovery. Stroke. (2016) 47:98. doi: 10.1161/STR.0000000000000098 27145936

[3] Milani G Antonioni A Baroni A Malerba P Straudi S. Relation between eeg measures and upper limb motor recovery in stroke patients: a scoping review. Brain Topogr. (2022) 35:651–66. doi: 10.1007/s10548-022-00915-y 36136166 PMC9684227

[4] Hordacre B Goldsworthy MR Welsby E Graetz L Ballinger S Hillier S. Resting state functional connectivity is associated with motor pathway integrity and upper-limb behavior in chronic stroke. Neurorehabil Neural Repair. (2020) 34:547–57. doi: 10.1177/1545968320921824 32436426

[5] Li W Li C Xiang Y Ji L Hu H Liu Y. Study of the activation in sensorimotor cortex and topological properties of functional brain network following focal vibration on healthy subjects and subacute stroke patients: an eeg study. Brain Res. (2019) 1722:146338. doi: 10.1016/j.brainres.2019.146338 31323197

[6] Limin S Yi W Dazhi Y Mingxia F Lili Z Yongshan H. Magnetic resonance imaging of active, passive and imaginary movement. Chin J Phys Med Rehabil. (2016) 38:126–31. doi: 10.3760/cma.j.issn.0254-1424.2016.02.011

[7] Xia W Dai R Xu X Huai B Bai Z Zhang J. Cortical mapping of active and passive upper limb training in stroke patients and healthy people: a functional near-infrared spectroscopy study. Brain Res. (1788) 2022:147935. doi: 10.1016/j.brainres.2022.147935 35500604

[8] Colombo R Sterpi I Mazzone A Delconte C Pisano F. Robot-aided neurorehabilitation in sub-acute and chronic stroke: does spontaneous recovery have a limited impact on outcome? NeuroRehabilitation. (2013) 33:621–9. doi: 10.3233/NRE-131002 24029005

[9] Dromerick AW Geed S Barth J Brady K Giannetti ML Mitchell A. Critical period after stroke study (cpass): a phase ii clinical trial testing an optimal time for motor recovery after stroke in humans. Proc Natl Acad Sci. (2021) 118:676118. doi: 10.1073/pnas.2026676118 PMC8488696 34544853

[10] Gao Z Pang Z Chen Y Lei G Zhu S Li G. Restoring after central nervous system injuries: neural mechanisms and translational applications of motor recovery. Neurosci Bull. (2022) 38:1569–87. doi: 10.1007/s12264-022-00959-x 36333482 PMC9723055

[11] Micera S Caleo M Chisari C Hummel FC Pedrocchi A. Advanced neurotechnologies for the restoration of motor function. Neuron. (2020) 105:604–20. doi: 10.1016/j.neuron.2020.01.039 32078796

[12] Thrane G Alt Murphy M Sunnerhagen KS. Recovery of kinematic arm function in well-performing people with subacute stroke: a longitudinal cohort study. J Neuroeng Rehabil. (2018) 15:67 doi: 10.1186/s12984-018-0409-4 30021596 PMC6052713

[13] Bernhardt J Hayward KS Kwakkel G Ward NS Wolf SL Borschmann K. Agreed definitions and a shared vision for new standards in stroke recovery research: the stroke recovery and rehabilitation roundtable taskforce. Int J Stroke. (2017) 12:444–50. doi: 10.1177/1747493017711816 28697708

[14] van den Heuvel MP Hulshoff Pol HE. Exploring the brain network: a review on resting-state fmri functional connectivity. Eur Neuropsychopharmacol. (2010) 20:519–34. doi: 10.1016/j.euroneuro.2010.03.008 20471808

[15] Bray S Arnold AE Levy RM Iaria G. Spatial and temporal functional connectivity changes between resting and attentive states. Hum Brain Mapp. (2015) 36:549–65. doi: 10.1002/hbm.22646 25271132 PMC6869123

[16] Cole MW Ito T Cocuzza C Sanchez-Romero R. The functional relevance of task-state functional connectivity. J Neurosci. (2021) 41:2684–702. doi: 10.1523/JNEUROSCI.1713-20.2021 33542083 PMC8018740

[17] Chen S Li Y Shu X Wang C Wang H Ding L. Electroencephalography mu rhythm changes and decreased spasticity after repetitive peripheral magnetic stimulation in patients following stroke. Front Neurol. (2020):11. doi: 10.3389/fneur.2020.546599 33133002 PMC7550716

[18] Kenzie JM Findlater SE Pittman DJ Goodyear BG Dukelow SP. Errors in proprioceptive matching post-stroke are associated with impaired recruitment of parietal, supplementary motor, and temporal cortices. Brain Imaging Behav. (2019) 13:1635–49. doi: 10.1007/s11682-019-00149-w 31218533

[19] Chen C Lee S Wang W Lin Y Su M. Eeg-based motor network biomarkers for identifying target patients with stroke for upper limb rehabilitation and its construct validity. PLoS One. (2017) 12:e178822. doi: 10.1371/journal.pone.0178822 PMC5470671 28614395

[20] Nasrallah FA Mohamed AZ Campbell ME Yap HK Yeow C Lim JH. Functional connectivity of brain associated with passive range of motion exercise: proprioceptive input promoting motor activation? Neuroimage (Orlando, Fla). (2019) 202:116023. doi: 10.1016/j.neuroimage.2019.116023 31325644

[21] Serrien DJ Strens LHA Cassidy MJ Thompson AJ Brown P. Functional significance of the ipsilateral hemisphere during movement of the affected hand after stroke. Exp Neurol. (2004) 190:425–32. doi: 10.1016/j.expneurol.2004.08.004 15530881

[22] Caliandro P Vecchio F Miraglia F Reale G Della MG La Torre G. Small-world characteristics of cortical connectivity changes in acute stroke. Neurorehabil Neural Repair. (2017) 31:81–94. doi: 10.1177/1545968316662525 27511048

[23] De Vico FF Pichiorri F Morone G Molinari M Babiloni F Cincotti F. Multiscale topological properties of functional brain networks during motor imagery after stroke. NeuroImage. (2013) 83:438–49. doi: 10.1016/j.neuroimage.2013.06.039 23791916

[24] Kim D Kim L Park W Chang WH Kim Y Lee S. Analysis of time-dependent brain network on active and mi tasks for chronic stroke patients. PLoS One. (2015) 10:e139441. doi: 10.1371/journal.pone.0139441 PMC4679158 26656269

[25] Qiu S Yi W Xu J Qi H Du J Wang C. Event-related beta eeg changes during active, passive movement and functional electrical stimulation of the lower limb. IEEE Trans Neural Syst Rehabil Eng. (2016) 24:283. doi: 10.1109/TNSRE.2015.2476481 26441422

[26] Wang H Xiong X Zhang K Wang X Sun C Zhu B. Motor network reorganization after motor imagery training in stroke patients with moderate to severe upper limb impairment. CNS Neurosci Ther. (2023) 29:619–32. doi: 10.1111/cns.14065 36575865 PMC9873524

[27] Bönstrup M Schulz R Schön G Cheng B Feldheim J Thomalla G. Parietofrontal network upregulation after motor stroke. Neuroimage: Clin. (2018) 18:720–9. doi: 10.1016/j.nicl.2018.03.006 29876261 PMC5987870

[28] Nolte G Bai O Wheaton L Mari Z Vorbach S Hallett M. Identifying true brain interaction from eeg data using the imaginary part of coherency. Clin Neurophysiol. (2004) 115:2292–307. doi: 10.1016/j.clinph.2004.04.029 15351371

[29] Rubinov M Sporns O. Weight-conserving characterization of complex functional brain networks. NeuroImage. (2011) 56:2068–79. doi: 10.1016/j.neuroimage.2011.03.069 21459148

[30] Lam TK Dawson DR Honjo K Ross B Binns MA Stuss DT. Neural coupling between contralesional motor and frontoparietal networks correlates with motor ability in individuals with chronic stroke. J Neurol Sci. (2018) 384:21–9. doi: 10.1016/j.jns.2017.11.007 29249372

[31] van Wijk BCM Litvak V Friston KJ Daffertshofer A. Nonlinear coupling between occipital and motor cortex during motor imagery: a dynamic causal modeling study. NeuroImage. (2013) 71:104–13. doi: 10.1016/j.neuroimage.2012.12.076 23313570

[32] Schulz R Koch P Zimerman M Wessel M Bönstrup M Thomalla G. Parietofrontal motor pathways and their association with motor function after stroke. Brain. (2015) 138:1949–60. doi: 10.1093/brain/awv100 25935722

[33] Cassidy JM Wodeyar A Wu J Kaur K Masuda AK Srinivasan R. Low-frequency oscillations are a biomarker of injury and recovery after stroke. Stroke. (2020) 51:1442–50. doi: 10.1161/STROKEAHA.120.028932 32299324 PMC7188582

[34] Ulanov M Shtyrov Y. Oscillatory beta/alpha band modulations: a potential biomarker of functional language and motor recovery in chronic stroke? Front Hum Neurosci. (2022) 16:940845. doi: 10.3389/fnhum.2022.940845 36226263 PMC9549964

[35] Zrenner C Kozak G Schaworonkow N Metsomaa J Baur D Vetter D. Corticospinal excitability is highest at the early rising phase of sensorimotor micro-rhythm. NeuroImage. (2023) 266:119805. doi: 10.1016/j.neuroimage.2022.119805 36513289

[36] Chen S Shu X Wang H Ding L Fu J Jia J. The differences between motor attempt and motor imagery in brain-computer interface accuracy and event-related desynchronization of patients with hemiplegia. Front Neurorobot. (2021):15. doi: 10.3389/fnbot.2021.706630 PMC8602190 34803647

[37] Sharma N Baron JC. Does motor imagery share neural networks with executed movement: a multivariate fmri analysis. Front Hum Neurosci. (2013) 7:564. doi: 10.3389/fnhum.2013.00564 24062666 PMC3771114

[38] Ogawa T Shimobayashi H Hirayama J Kawanabe M. Asymmetric directed functional connectivity within the frontoparietal motor network during motor imagery and execution. NeuroImage. (2022) 247:118794. doi: 10.1016/j.neuroimage.2021.118794 34906713

[39] Shim M Choi G Paik N Lim C Hwang H Kim W. Altered functional networks of alpha and low-beta bands during upper limb movement and association with motor impairment in chronic stroke. Brain Connect. (2021). doi: 10.1089/brain.2021.0070 34269616

[40] Wang L Yu C Chen H Qin W He Y Fan F. Dynamic functional reorganization of the motor execution network after stroke. Brain. (2010) 133:1224–38. doi: 10.1093/brain/awq043 20354002

[41] Xin X Duan F Kranz GS Shu D Fan R Gao Y. Functional network characteristics based on eeg of patients in acute ischemic stroke: a pilot study. Neurorehabilitation. (2022) 51:455–65. doi: 10.3233/NRE-220107 35848041

